# Supplementary material for: Sequence-specific dynamic DNA bending explains mitochondrial TFAM’s dual role in DNA packaging and transcription initiation
Source: Nat Commun. 2024 Jun 27;15:5446. doi: 10.1038/s41467-024-49728-6 (PMC11211510; doi:10.1038/s41467-024-49728-6)
Supplement: Supplementary file 1 — Supplementary Information [file 41467_2024_49728_MOESM1_ESM.pdf]

Supplementary Information

Sequence-specific dynamic DNA bending by mitochondrial transcription factor TFAM explains its dual role in DNA packaging and transcription initiation

Hyun Huh[1,#], Jiayu Shen[2,3,#], Yogeeshwar Ajjugal[3], Aparna Ramachandran[3], Smita S Patel[3,*] and Sang-Hyuk Lee[1,4,*]

[1]Institute for Quantitative Biomedicine, Rutgers University, Piscataway, New Jersey 08854, USA

[2]Graduate School of Biomedical Sciences, Robert Wood Johnson Medical School, Rutgers University, Piscataway, New Jersey 08854, USA

[3]Department of Biochemistry and Molecular Biology, Robert Wood Johnson Medical School, Rutgers University, Piscataway, New Jersey 08854, USA

[4]Department of Physics and Astronomy, Rutgers University, Piscataway, New Jersey 08854, USA

[*]Correspondence: patelss@rutgers.edu and shlee@physics.rutgers.edu

[#]These authors contributed equally

Supplementary Note

Sequence analysis of LSP, HSP, and NS

Because we currently lack sufficient information, it is difficult to pinpoint the molecular origins of the sequence-dependent bending dynamics of DNA-TFAM complex among LSP, HSP, and NS; particularly, why LSP induces a more stable, fully bent state than HSP and NS sequences. Nevertheless, the basic sequence analysis and comparison of LSP, HSP, and NS, as detailed below, provide some clues.

A recent study [1] pointed out that a $GN_{10}G$ sequence motif is required for high-affinity TFAM binding. This motif is present in LSP, HSP, and the NS DNA that was used in our studies (Supplementary Fig. 7, yellow highlight). Our analysis indicates the pair of guanines in the $GN_{10}G$ motif of LSP contains 5'-TG-3'/5'-CA-3' dinucleotide steps (in red, Supplementary Fig. 7), which are more deformable base steps according to literature [2-4]. Previously published data pointed out that TG/CA dinucleotide steps act as a better target site for high mobility group proteins [5].

Further, we also found that adjacent to these 5'-TG-3'/5'-CA-3' steps, there are 5'-TT-3'/5'-AA-3' steps present in the LSP (green box, Supplementary Fig. 7). This AT-rich sequence provides higher flexibility (due to low base stacking energy) and creates a perfect kink that can stabilize the bend conformation [6]. Unlike LSP, the HSP lacks one of the bona fide 5'-TG-3'/5'-CA-3' steps at the downstream end and lacks the TT/AA dinucleotide step around the TG/CA step. The NS sequence contains a $GN_{10}G$ motif (scheme 1, Supplementary Fig. 7) but lacks the bona fide TG/CA motif and the TT/AA dinucleotide step (scheme 2, Supplementary, Fig. 7). Published structural studies show additional base contacts in the TFAM-LSP complex that are absent in HSP-TFAM and NS-TFAM complexes [7].

Additionally, LSP has an A…T rich downstream end sequence compared with the HSP and NS sequence (dashed brown box, Figure S7), which may stabilize the fully bent state. Previous studies stated that the CA/TG dinucleotide step in the 5' end of the oligo A tract strongly modulates the intrinsic DNA bendability [8].

Finally, the stacking energy of each dinucleotide step in LSP, HSP, and NS sequences gives us a clue about the overall flexibility of these sequence [6]. The LSP has the lowest average stacking

energy of all the dinucleotide steps (-7.27 kcal/mol), followed by NS (-8.12 kcal/mol) and HSP (-8.33 kcal/mol).

This analysis creates testable hypotheses. However, testing these hypotheses will require a systematic examination of a series of DNA sequences (mutating the TG/CA and TT/AA dinucleotide steps) coupled with smFRET studies to measure the precise DNA bending dynamics.

Supplementary Figures

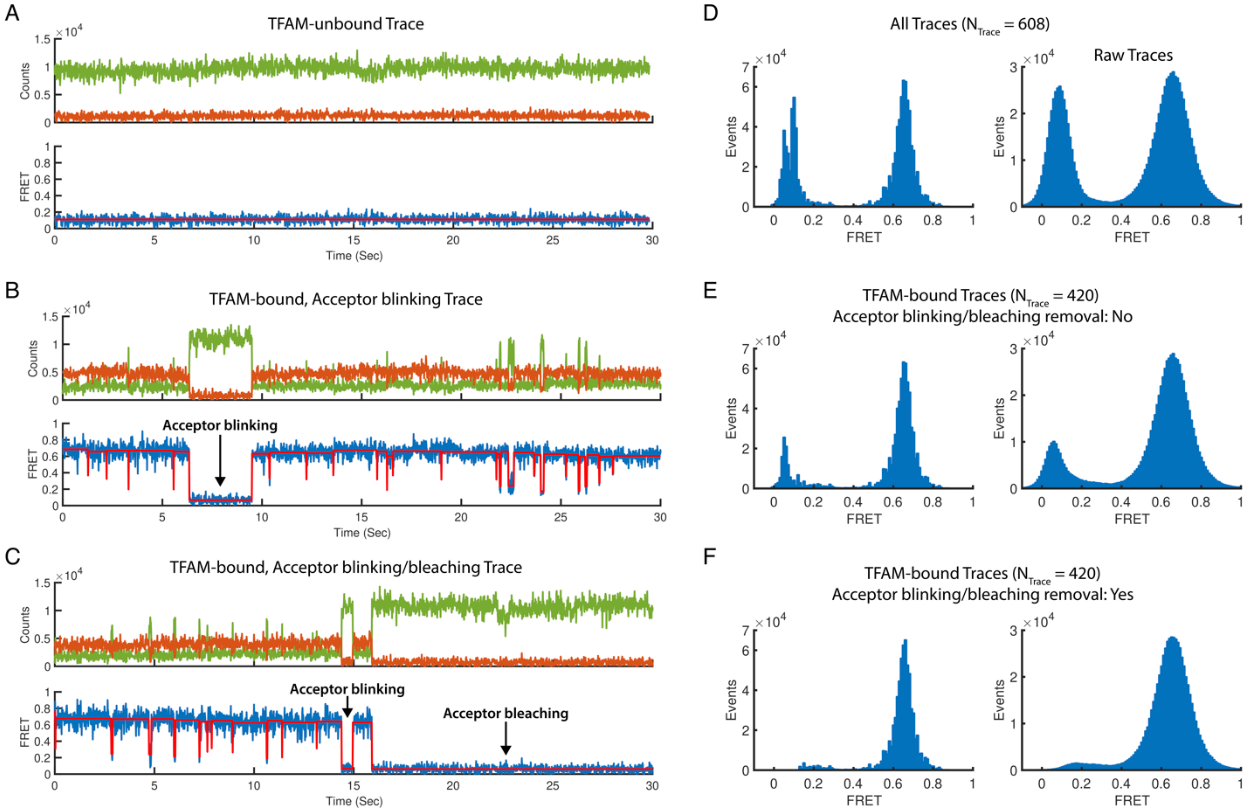

Supplementary Fig. 1. [Identification and removal of smFRET signals arising from TFAM-unbound DNA and photobleached dyes], Related to Figure 1

Single-molecule FRET data include time traces of various situations irrelevant to DNA-TFAM interaction, such as (A) TFAM-unbound and (B-C) acceptor fluorescence blinking/bleaching. Including all these irrelevant data obscures the signature of the transient P-state. Hidden Markov Model fitting of smFRET traces using vbFRET or ebFRET MATLAB software packages enables distinguishing the P-state from TFAM-unbound or acceptor-bleached situations based on the fitted FRET values. (D-F) FRET histograms of fitted traces (left panel) and raw traces (right panel): (D) when all traces are included; (E) when TFAM-unbounded traces are excluded; and (F) acceptor blinking/bleaching events are additionally excluded.

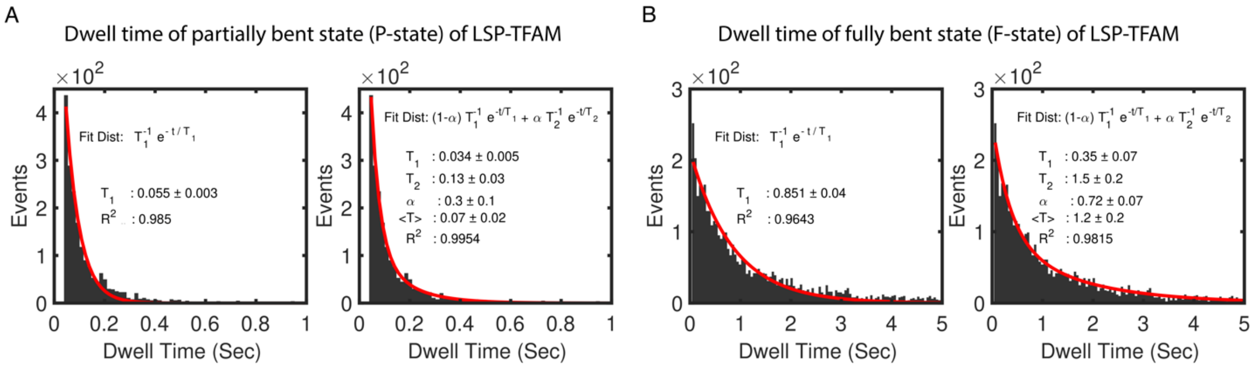

Supplementary Fig. 2. [Comparison of single- and double-exponential model fitting of the P- and F-state dwell time distributions of the LSP-TFAM complex], Related to Figure 1

(A) Single-exponential function fitting of the P-state (left panel) and F-state (right-panel) dwell time distribution. (B) Double-exponential function fitting of the P-state (left panel) and F-state (right-panel) dwell time distribution. The F-state dwell time distribution is fitted poorly to single-exponential models but is fitted well to a double-exponential model with a mean dwell time of 1.2 (± 0.2) sec. In contrast, the P-state dwell time distribution is fitted to a single-exponential model with a mean dwell time of 0.055 (± 0.003) fairly well, although double-exponential models slightly improve the fitting, especially in the long tail region of the distribution. The +/- values inside parenthesis represent the uncertainty in the fitted parameters, which are calculated from the 95% confidence intervals of exponential fitting performed by MATLAB 'fit' function.

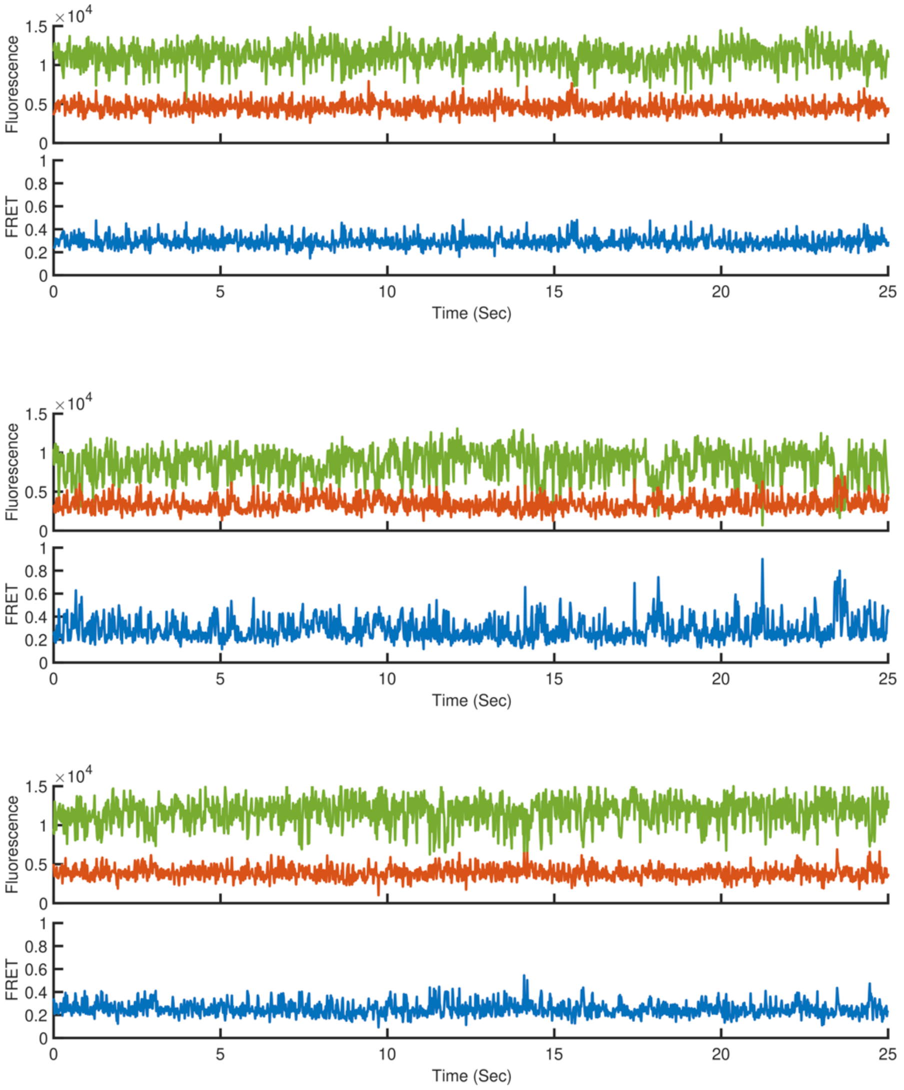

Supplementary Fig. 3. [More smFRET trace samples of C-tail mutant TFAM (TFAM-ΔC26) bound to LSP], Related to Figure 1

Three additional sample traces here showcase frequent but failed attempts of the C-tail deleted TFAM to fully bend LSP DNA, which contribute to the broad FRET distribution in Figure 1G.

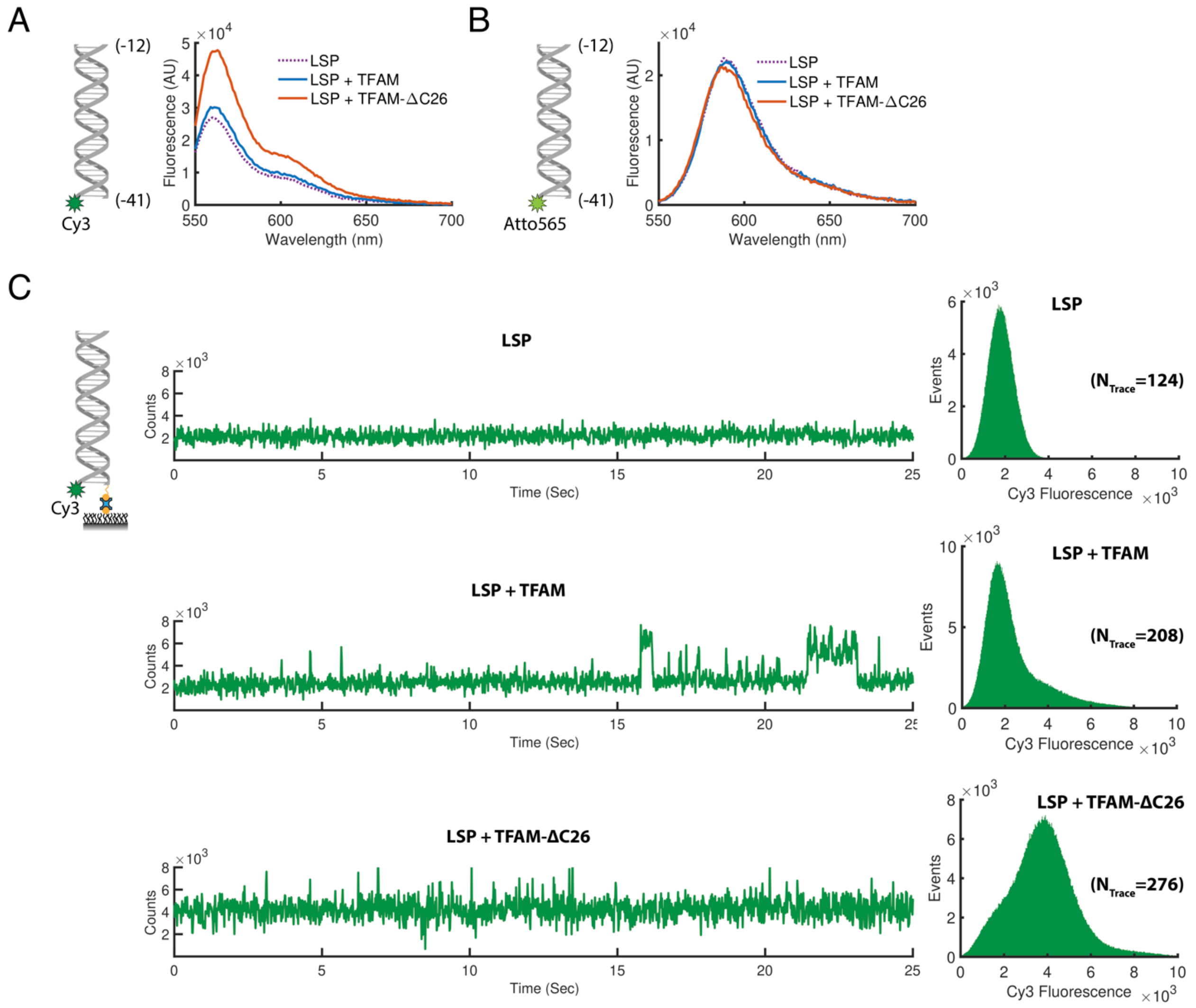

Supplementary Fig. 4. [Supplementary data about PIFE effect by WT and C-tail mutant TFAM (TFAM-ΔC26) bound to Cy3-labeld LSP], Related to Figure 2

(A) Ensemble fluorescence emission spectra of Cy3-labeled LSP DNA show PIFE effects upon TFAM binding. (B) In contrast, the PIFE effects disappear when Atto565-labeled DNA is used instead. (C) Single-molecule Cy3 fluorescence time traces and histograms of LSP alone (upper panel) and upon addition of WT TFAM (middle panel) or C-tail mutant TFAM (lower panel). Figures 2H-2J are duplicated here for convenience.

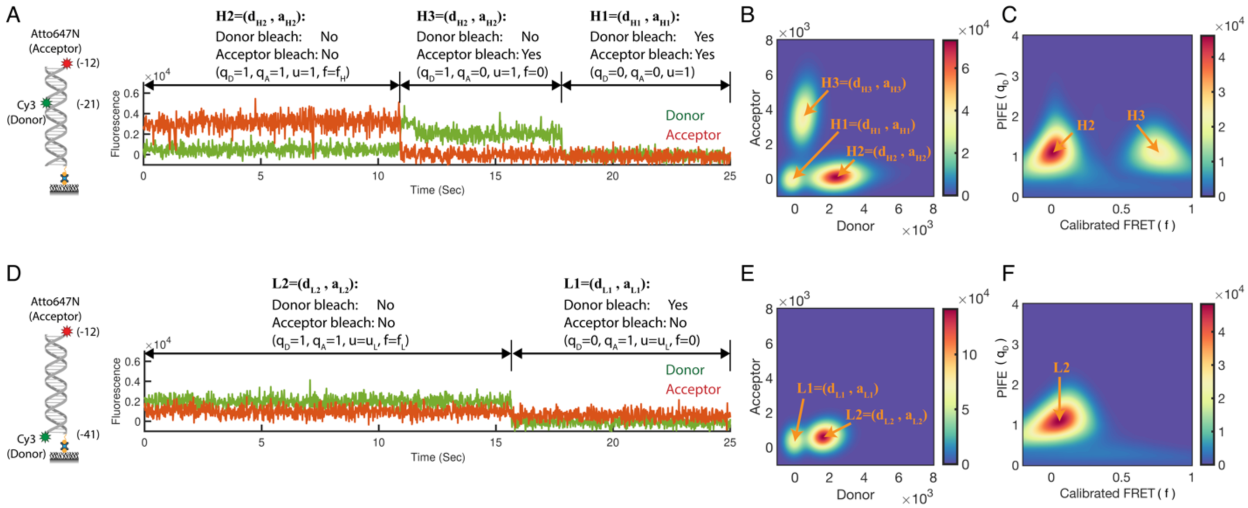

Supplementary Fig. 5. [Donor/acceptor detection channel calibration to extract PIFE and FRET factors from single-molecule fluorescence traces of Cy3- and Atto647N-labeled DNA], Related to Figure 2

(A-B) LSP DNA end-labeled with Atto647N and mid-labeled with Cy3 provides instrument-specific donor and acceptor fluorescence for three situations: a high FRET, donor only (i.e., acceptor bleaching), and no fluorophore (i.e., both donor and acceptor bleaching), which are symbolized by H2, H3, and H1, respectively. (D-E) Similar LSP DNA with end-labeled Cy3 instead provides donor and acceptor fluorescence for two additional situations: a low FRET, and acceptor only (i.e., donor bleaching), which are symbolized by L2, and L1, respectively. The representative donor and acceptor fluorescence values (d_{H2}, a_{H2}), (d_{H3}, a_{H3}), (d_{H1}, a_{H1}), (d_{L2}, a_{L2}), and (d_{L1}, a_{L1}) corresponding to the five situations can be estimated by 2-D Gaussian mixture model clustering of the 2-D donor/acceptor scatter plots (B, E), which then can be used for calibrating the fluorescence detection channels of a specific instrument (see Methods). (C, F) Applying the calibration back to the donor/acceptor time traces enable to extract calibrated FRET and PIFE factors (see Methods). The PIFE factor (i.e., normalized quantum efficiency of donor emission) shows ~1 regardless of FRET values in case of LSP DNA alone as expected. However, the same calibration result herein demonstrated increased PIFE factor upon addition of TFAM as shown in Figure 2F and 2L in the main text. The variables q_D, q_A, u, and f represent normalized quantum efficiency (QE) of donor emission by donor-excitiation light, normalized QE of acceptor emission by donor-excitation light, normalized donor-excitation power, and calibrated FRET, respectively (see Methods). f_H implies the representative values of f for H2 event; and f_L and u_L implies the representative values of f and u for L2 event.

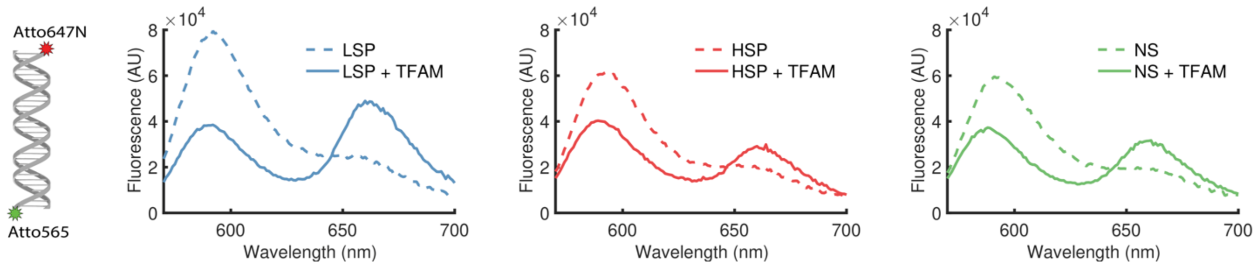

Supplementary Fig. 6. [Ensemble fluorescence spectra of Atto565 and Atto647N labeled DNA], Related to Figure 4

The fluorescence emission signal was measured in 570-700 nm wavelength range with 1 nm increment under 561 nm excitation. Donor (Atto565) fluorescence depends on the sequence of DNA to which the dye is labeled, LSP showing stronger signal than other DNA. To better visualize the sequence-dependence of ensemble FRET in the presence of TFAM, the total signal in the 570-700 nm spectral range was normalized to 100% for each DNA in Figure 4K. The ensemble FRET efficiency values were more rigorously calculated (see Method details) and were presented in Figure 4L.

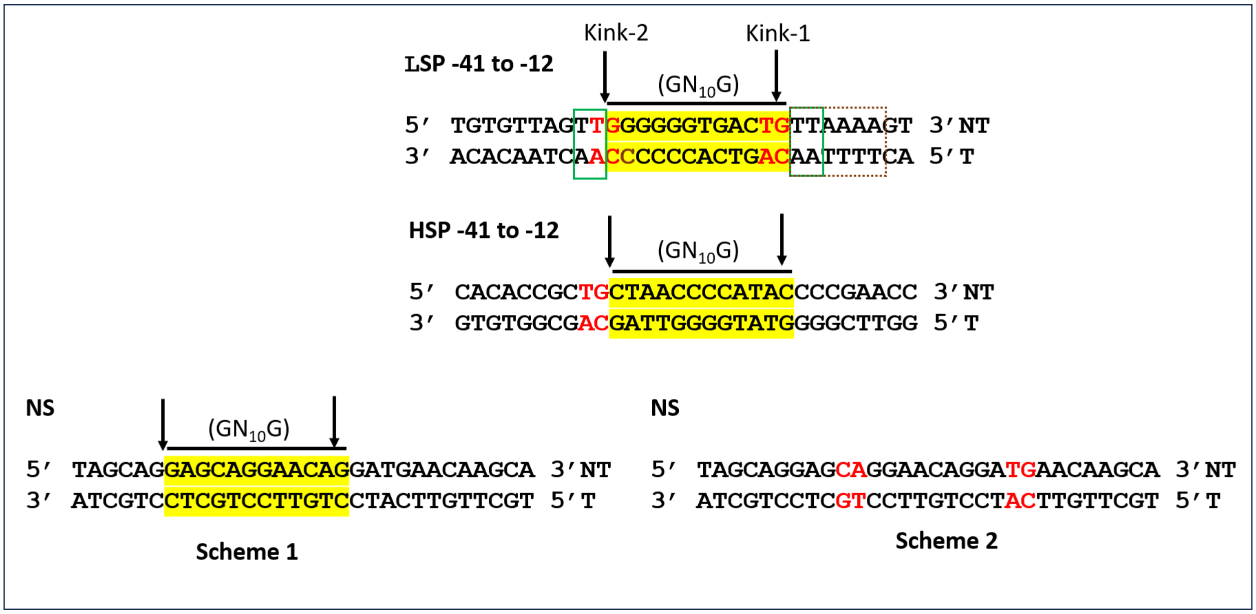

Supplementary Fig. 7. [Comparative sequence analysis of LSP, HSP and NS]

See 'Sequence analysis of LSP, HSP, and NS' section of Supplementary Texts and Supplementary References for the details. Note that for NS sequence there is no structural data available, so the predicted kink sites are based on $GN_{10}G$ sequence motif.

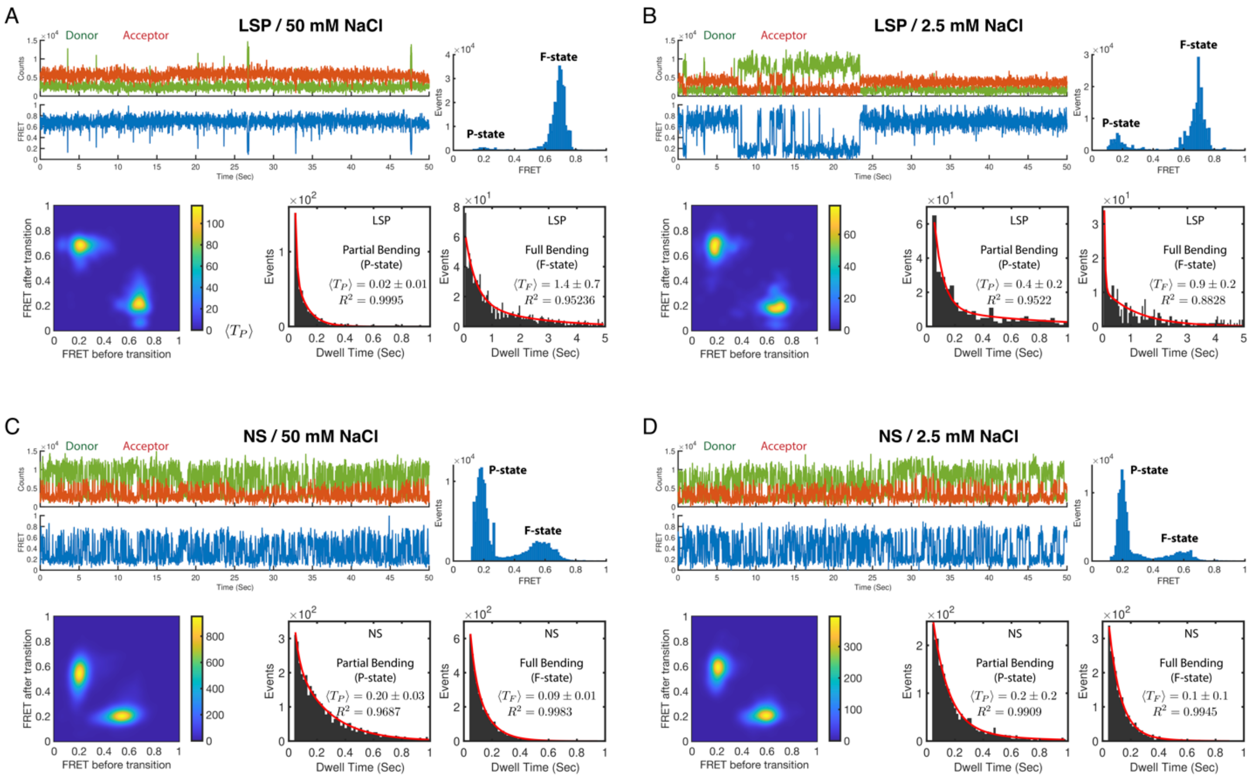

Supplementary Fig. 8. [Effects of NaCl concentration on DNA-TFAM bending dynamics]

(A-B) LSP-TFAM bending dynamics measured from single-molecule FRET (smFRET) data for two different NaCl concentrations, 50 mM (A) and 2.5 mM (B). Shown In each subfigure are a representative single-molecule time trace, a FRET histogram, a transition density plot (TDP), and the P-/F-state dwell time distributions with double-exponential fit curves. Whereas LSP-TFAM shows a high stability in the fully bent F-state at 50 mM NaCl, lowering NaCl concentration to 2.5 mM introduces time intervals when the F-state is unstable. This results in substantial increase of the mean P-state dwell time from 0.02 sec to 0.4 sec, while decreasing the mean F-state dwell time from 1.4 sec to 0.9 sec in this dataset. (C-D) NS-TFAM bending dynamics for 50 mM (C) and 2.5 mM (D) NaCl, showing insensitivity to NaCl concentration. N_{Trace}: 102 (A), 83 (B), 66 (C), 53 (D). smFRET assay and analysis applied here is identical to what was used for Figure 1 and 3.

Supplementary Tables

$$D + A \underset{\tilde{k}_{off}}{\overset{k_{on}}{\rightleftharpoons}} (DA)_P \underset{k_{F\to P}}{\overset{k_{P\to F}}{\rightleftharpoons}} (DA)_F$$

	Figure Ref.	LSP	HSP	NS
k_{on} ($min^{-1} \cdot nM^{-1}$)	4D, 4H	0.055 (± 0.028)	0.157 (± 0.065)	0.171 (± 0.047)
$\tilde{k}_{off}$ (min^{-1})	4D	12.2 (± 4.4)	18.1 (± 5.1)	11.9 (± 2.1)
$\tilde{K}_d(= \tilde{k}_{off}/k_{on})$ (nM)	4H	223 (± 79)	115 (± 35)	69 (± 14)
$Mean(FRET_P)$	3H	0.219 (± 0.014)	0.267 (± 0.011)	0.205 (± 0.005)
$Mean(FRET_F)$	3H	0.647 (± 0.016)	0.509 (± 0.040)	0.480 (± 0.045)
$Std(FRET_P)$	3I	0.0393 (± 0.0087)	0.0463 (± 0.0087)	0.0230 (± 0.0026)
$Std(FRET_F)$	3I	0.041 (± 0.011)	0.0417 (± 0.0067)	0.0667 (± 0.0032)
$\langle T_P \rangle$ $(= 1/k_{P\to F})$ (sec)	3J	0.042 (± 0.011)	0.045 (± 0.008)	0.116 (± 0.027)
$\langle T_F \rangle$ $(= 1/k_{F\to P})$ (sec)	3J	1.01 (± 0.30)	0.073 (± 0.020)	0.065 (± 0.014)
$K_{F/P}$ $(= \langle T_F \rangle / \langle T_P \rangle)$	3K	24.6 (± 7.6)	1.70 (± 0.74)	0.59 (± 0.24)

Supplementary Table 1. [Summary of DNA-TFAM interaction kinetic model parameters]

All experiments were performed in triplicate, and the mean and standard deviation of three samples are shown here for each parameter. The parameters about the two conformational states, P- and F-state, and their kinetics were obtained from single-molecule FRET (smFRET) data. $Mean(FRET_P)$, $Mean(FRET_F)$, $Std(FRET_P)$, and $Std(FRET_F)$ represent the centers and the widths of the two primary clusters shown in FRET transition density plots (TDP) (see Figure 1E, 3B, 3E), and they were estimated by 2-D Gaussian mixture model fitting. The mean dwell time $\langle T_P \rangle$ and $\langle T_F \rangle$ in the P- and F-state were obtained by fitting the dwell time distributions with double-exponential functions for LSP and single-exponential functions for HSP and NS. The bending/unbending equilibrium constant $K_{F/P}$ was obtained from $\langle T_P \rangle$, $\langle T_F \rangle$, and the formula

$K_{F/P} = \langle T_F \rangle / \langle T_P \rangle$. DNA-TFAM dissociation rate $\tilde{k}_{off}$ and equilibrium dissociation constant $\tilde{K}_d$ were obtained from ensemble FRET-based stopped-flow assay and ensemble fluorescence anisotropy-based assay, respectively. The association constant k_{on} was calculated from the $\tilde{k}_{off}$, $\tilde{K}_d$, and the formula $\tilde{K}_d = \tilde{k}_{off}/k_{on}$. The +/- values inside parenthesis represent the uncertainty in the estimated parameters, which are calculated from the confidence intervals of exponential fitting performed on each of the three independent experiments for each condition and error propagation rules. The exponential fitting and the evaluation of confidence interval were performed by MATLAB 'fit' function.

Supplementary References

1. Choi, W.S. and M. Garcia-Diaz, *A minimal motif for sequence recognition by mitochondrial transcription factor A (TFAM).* Nucleic Acids Research, 2022. **50**(1): p. 322-332.
2. Travers, A.A., *DNA bending and kinking—sequence dependence and function: Current Opinion in Structural Biology 1991, 1: 114–122.* Current Opinion in Structural Biology, 1991. **1**(1): p. 114-122.
3. Olson, W.K. and V.B. Zhurkin, *Working the kinks out of nucleosomal DNA.* Current opinion in structural biology, 2011. **21**(3): p. 348-357.
4. Ojha, R.P., et al., *DNA bending and sequence‐dependent backbone conformation: NMR and computer experiments.* European journal of biochemistry, 1999. **265**(1): p. 35-53.
5. Churchill, M., et al., *HMG‐D is an architecture‐specific protein that preferentially binds to DNA containing the dinucleotide TG.* The EMBO Journal, 1995. **14**(6): p. 1264-1275.
6. Ussery, D.W., *DNA Structure: A-, B-and Z-DNA Helix Families.* Encyclopedia of life sciences, 2002. **1**: p. e003122.
7. Ngo, H.B., et al., *Distinct structural features of TFAM drive mitochondrial DNA packaging versus transcriptional activation.* Nature communications, 2014. **5**(1): p. 3077.
8. Nagaich, A.K., et al., *CA/TG sequence at the 5'end of oligo (A)-tracts strongly modulates DNA curvature.* Journal of biological chemistry, 1994. **269**(10): p. 7824-7833.
