# Supplementary material for: Sequence-specific dynamic DNA bending explains mitochondrial TFAM’s dual role in DNA packaging and transcription initiation
Source: Nat Commun. 2024 Jun 27;15:5446. doi: 10.1038/s41467-024-49728-6 (PMC11211510; doi:10.1038/s41467-024-49728-6)
Supplement: Supplementary file 3 — Description of Additional Supplementary Files [file 41467_2024_49728_MOESM3_ESM.pdf]

Description of Additional Supplementary Files

Supplementary Software 1: Instruction manual of the custom software that was devel oped for the analysis of smFRET and smPIFE data.
