# Supplementary material for: Sequence-specific dynamic DNA bending explains mitochondrial TFAM’s dual role in DNA packaging and transcription initiation
Source: Nat Commun. 2024 Jun 27;15:5446. doi: 10.1038/s41467-024-49728-6 (PMC11211510; doi:10.1038/s41467-024-49728-6)
Supplement: Supplementary file 4 — Supplementary Software 1 [file 41467_2024_49728_MOESM4_ESM.pdf]

# ‘smfretpostanalypub’ (smFRET Analysis Software) Instruction Manual

(https://github.com/shyuklee/smfretpostanalypub)

Sang-Hyuk Lee

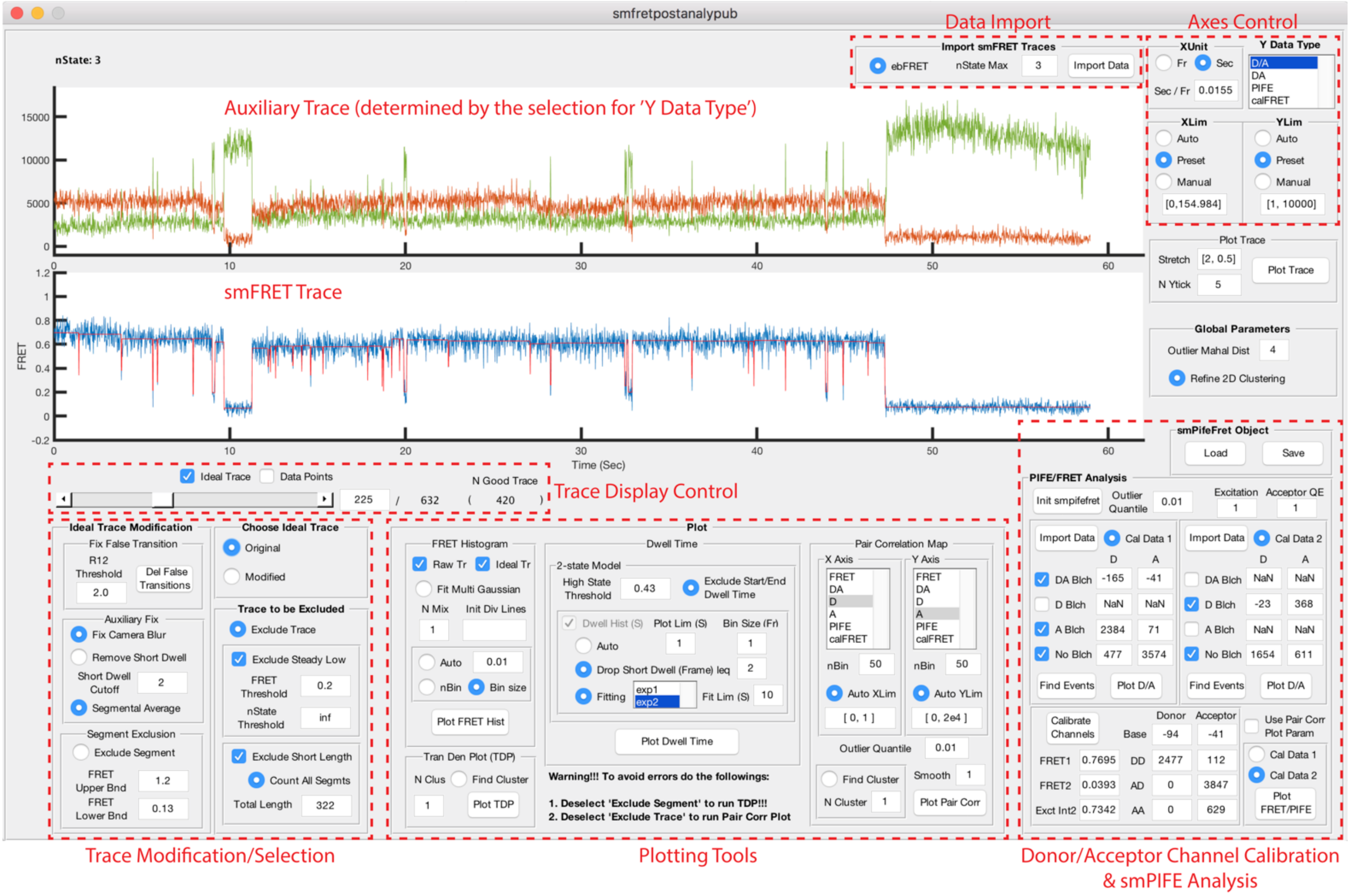


## Introduction

‘smfretpostanalypub’ imports single-molecule FRET (smFRET) trace data and performs various analysis and plotting. The current public release (v1.0) can import MATLAB MAT-files saved from ebFRET. This software was developed using MATLAB_R2020a and was packaged into a standalone application for 64-bit Mac OSX (arch: ‘maci64’) and Windows (arch: ‘win64’) systems. A screenshot of the software is shown above. Each task is grouped inside a panel with a self-explanatory title. Two main graph windows display smFRET trace (lower window) and auxiliary

trace (upper window) such as donor/acceptor signals. Different types of auxiliary trace can be selected by *'Y Data Type'* list box. As indicated by dotted red rectangular boxes in the screenshot, the tasks are largely classified as Data Import; Trace Display Control; Axes Control; Trace Modification/Selection; Plotting Tools; and Donor/Acceptor Channel Calibration & smPIFE Analysis. Each task will be explained in more detail below.

## Install

A single executable file (smfretpostanalypub.app for Mac and smfretpostanalypub.exe for Windows) can be placed in any folder. The software recognizes the current working folder as the default location for file input/output. It is therefore recommended to put the executable file where smFRET trace data is located for convenience. **(Note: the current working folder path name should not contain a blank space for Mac OSX; otherwise, the default file IO folder is reset to the system root folder.)**

**Prerequisites:** Version 9.8 (R2020a) of the MATLAB Runtime (MCR) should be installed. If you have MATLAB installed, MCR may have been already installed too. To check it, enter

```
>> mcrinstaller
```

at the MATLAB prompt. Even if the version of the installed MCR is different than 9.8, the software might still work. Simply run smfretpostanalypub.app or smfretpostanalypub.exe to see if any MCR-related error is invoked. Even if you don't have MATLAB, you can still run the software by downloading and installing MCR V9.8 from the following link on the MathWorks website:

https://www.mathworks.com/products/compiler/mcr/index.html

More information about MCR can be found from the following link:

https://www.mathworks.com/help/compiler/matlab-runtime.html

Download and installation of MCR will take less than 5 min on a typical desktop computer.

## Description of software

**Prerequisites:** Raw smFRET data are typically acquired as time-lapse images with a Total Internal Reflection Fluorescence (TIRF) microscope and an Electron Multiplying CCD (EMCCD) camera. The analysis of raw image data to identify bright fluorescence spots as single molecules and track their donor/acceptor fluorescence time traces can be performed with freely available software developed by several research groups (e.g., https://github.com/Ha-

SingleMoleculeLab). In the next step, smFRET traces constructed from donor/acceptor time traces are then fitted with Hidden Markov Models (HMM) to characterize the dynamic transitions of smFRET using several publicly available software: HaMMY, vbFRET, and ebFRET. The software smfretpostanalypub herein performs additional analysis and plotting of HMM-fitted trace data. The major functions of the software are explained below.

**Data Import:** smfretpostanalypub currently supports only MATLAB MAT-file generated by ebFRET. *'Import Data'* button opens a window for users to select ebFRET files. Multiple files can be selected to analyse them all.

**Trace Display Control:** A specific trace to be displayed can be chosen by either changing the slider position or directly entering a trace ID in the text box. Display of the HMM-fitted trace can be toggled by *'Ideal Trace'* check box. The total number of imported traces is shown. The number of good traces after trace screening is also displayed in *'N Good Trace'* text box.

**Axes Control:** The time unit (frame or second) and the display range of x- and y-axis can be changed here. The data type displayed in the auxiliary trace window can be selected among donor/acceptor (D/A), donor-acceptor signal sum (DA), smPIFE trace (PIFE), and calibrated smFRET trace (calFRET) in *'Y Data Type'* list box.

**Trace Modification/Selection:** The ideal traces fitted by ebFRET can be further modified by a set of analyses within *'Ideal Trace Modification'* panel.
*Fix False Transition:* For traces of high noise level, it is challenging to distinguish transition events of short-lived states from noise. If the user deems the HMM-fitting to be prone to identify noise as state-transitions, the transition events can be more conservatively assigned by running the analysis in this panel. The algorithm takes two adjacent smFRET trace segments that are identified as different states; computes their mean and standard deviation $(\mu_1, \sigma_1)$ and $(\mu_2, \sigma_2)$; computes $R_{12} = |\mu_1 - \mu_2|/\sqrt{\sigma_1^2 + \sigma_2^2}$ ; and compares $R_{12}$ to the value in *'R12 Threshold'* text box. If $R_{12}$ is smaller than the threshold, the two segments are merged. *'Del False Transitions'* button initiates the process to generate modified ideal traces. *'Choose Ideal Trace'* panel allows switching between original and modified traces.
*Auxiliary Fix:* It performs additional modification of ideal traces. During a transition between two FRET states, often times both the states partially reside within a single camera frame, producing an intermediate FRET value. HMM-fitting identifies it as a separate state sometime. *'Fix Camera*

*Blur'* forces the intermediate FRET state to be absorbed into the nearest neighboring state (i.e., either the lower or the higher FRET state). Similarly, *'Remove Short* Dwell' allows the user to also remove any state with dwell time shorter than the value in *'Short Dwell Cutoff'* text box. In HMM-fitting, a segment of trace is forcefully assigned to one of a certain number of states (i.e., 3 states). HMM-fitted ideal traces usually well identify the segments of different FRET states, but sometime the actual FRET value of a segment appears different from the one assigned by HMM-fitting. *'Segmental Average'* maintains the segments of FRET states but changes the FRET values of HMM-fitted ideal states to the segment-wise average of raw FRET traces.
<u>*Segment Exclusion:*</u> *'Exclude Segment'* allows the user to exclude segments of trace, whose FRET values are outside a certain range (defined by *'FRET Upper Bnd'* and *'FRET Lower Bnd'* text boxes), from further analysis. This is useful for removing the events of acceptor photo-bleaching and/or photo-blinking.
<u>*Trace to be Excluded:*</u> The user can select certain traces for analysis based on several criteria in this panel. *'Exclude Steady Low'* check box removes the ideal trace if the number of states is equal or smaller than the value in *'nState Threshold'* text box and the FRET values are smaller than the threshold value *in 'FRET Threshold'* text box. This is useful for eliminating traces that represent absence of biophysical activity (e.g., protein-unbound DNA). Donor/acceptor photo-bleaching and photo-blinking shorten the length of traces that is eligible for analysis. *'Exclude Short Length'* check box enables the user to exclude such traces with eligible length shorter than the threshold frame number in *'Exclude Short Length'* text box.

**Plotting Tools:** Four types of analysis/plotting can be performed: FRET Histogram, Transition Density Plot (TDP), Dwell Time Analysis, and Pair Correlation Map.
<u>*FRET Histogram:*</u> FRET histograms of raw and/or ideal traces can be selected with *'Raw Tr'* and *'Ideal Tr'* check boxes. *'Fit Multi Gaussian'* button fits FRET histograms with 1D Gaussian mixture models. The user can provide the number of Gaussian populations and the initial guess of FRET values dividing the populations to *'N Mix'* and *'Init Div Lines'* text boxes, respectively. The histogram bin size can be determined either automatically (*'Auto'* button) or manually (*'nBin'* or *'Bin size'* button). The value in the text box represents either number of bins or bin size in the manual mode, depending on whether *'nBin'* or *'Bin size'* button is selected.
<u>*Tran Den Plot (TDP):*</u> *'Plot TDP'* button generates TDP **(Note: *'Exclude Segment'* button should be unchecked when plotting TDP; otherwise, the plot will appear blank)**. The major populations appearing in TDP can be localized with 2D Gaussian mixture models-based

clustering algorithms if *'Find Cluster'* button is checked. The number of clusters should be provided by the user in 'N Clus' text box.

Dwell Time: Various methods to estimate kinetic rates of transitions among FRET states have been published. These methods become quite sophisticated when the number of states gets large and they are typically integrated in HMM-fitting. Because smfretpostanalypub modifies HMM-fitted ideal traces through postprocessing in a way the HMM-defined states are invalidated, a rather robust way to estimate the kinetic rates through dwell time analysis (currently works only for 2-state models) is implemented here. The intervals of states in ideal traces are reassigned to either high or low FRET states based on the user-provided threshold value in *'High State Threshold'* text box, which can be reasonably determined from the FRET histogram plot. The dwell time data in the beginning and the end of a trace can be excluded from dwell time analysis with checking *'Exclude Start/End Dwell Time'* text box. *'Plot Dwell Time'* button plots dwell time histograms of the low and the high FRET states. The histogram plot range and bin size are automatically determined if *'Auto'* button is checked but otherwise they are manually provided by the values in *'Plot Lim (S)'* and *'Bin Size (Fr)'* text boxes. A dwell time histogram follows a single exponential decay function in the case of first-order kinetics, but often times a weighted sum of two exponential decay functions better fit smFRET data when two degenerate states with almost identical FRET values are present. The fitting of dwell time histogram with single or double exponential functions can be performed with *'Fitting'* button and *'exp1 vs exp2'* fit-model choice list box. Dwell time longer than the value in *'Fit Lim (S)'* text box is considered as outliers and is discarded. Events faster than camera frame rate are beyond the detection limit of a microscope. The degraded detection efficiency for short dwell time relative to camera frame rate makes dwell time histograms deviate from exponential distribution functions at short dwell time range; hence, dropping short dwell time data improves the precision of exponential function fitting, which is implemented by *'Drop Short Dwell (Frame) leq'* button and text box.

Pair Correlation Map: This panel is for plotting pairwise 2D heat map (i.e., smoothed 2D histogram) of any choice of two quantities. **(Note: *'Exclude Trace'* button should be unchecked when plotting pairwise 2D heat map; otherwise, errors will be invoked).** *'X Axis'* and *'Y Axis'* list boxes display the supported quantities to be selected by the user, where D, A, and DA stand for donor, acceptor, and donor-acceptor sum, respectively. *'nBin'* text boxes specify number of bins in X- and Y-axis. The range of plot can be automatically determined based on the outlier quantile value in '*Outlier Quantile'* text box or the user can provide them in the form of '[lower bound, upper bound]' inside the text boxes located below *'Auto XLim'* and *'Auto YLim'* buttons. The major populations appearing in 2D heat map can be localized with 2D Gaussian mixture

models-based clustering algorithms if *'Find Cluster'* button is checked. The number of clusters should be provided by the user in 'N Cluster' text box.

**Donor/Acceptor Channel Calibration & smPIFE Analysis:** This panel allows the user to calibrate donor and acceptor fluorescence detection channels in order to generate correlative smPIFE-smFRET traces when Cy3 is used as donor fluorophore. The theoretical basis and experimental implementation are explained in Method section and Fig. S5. The detailed procedure of how to perform channel calibration and smPIFE analysis is explained in the last subsection of Example Usage below.

## Example Usage

### smFRET trace data import

1. Click on *'Import Data'* inside '*Import smFRET Traces'* panel.
2. Go to sample_trace > LSP_Atto565_Atto647 folder to choose four MAT-files, representing ebFRET-processed smFRET traces for Atto565/Atto647-labeled LSP DNA in the presence of TFAM protein.

### FRET histogram plot (reproduce Fig. S1D, E, and F)

1. Fig. S1D shows FRET histograms for all the traces of length longer than 5 sec. To reproduce it, configure the parameters as shown in the screenshot on the right.

   *'Trace to be Excluded'* panel:

   >> *'Exclude Trace'* = True

   >> *'Exclude Steady Low'* = False

   >> *'Exclude Short Length'* = True

   >> *'Total Length'* = 322

   *'Segment Exclusion'* panel:

   >> *'Exclude Segment'* = False

   *'FRET Histogram'* panel:

   >> *'Raw Tr'* = True; *'Ideal Tr'* = True

   >> *'Bin Size'* = 0.01

   >> Click on *'Plot FRET Hist'* button

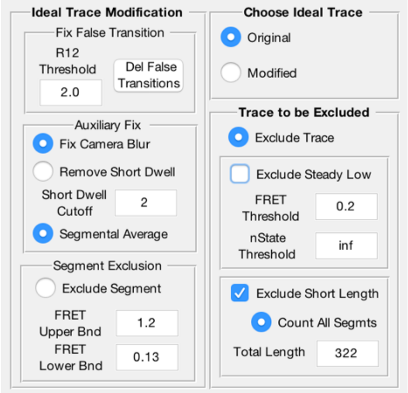


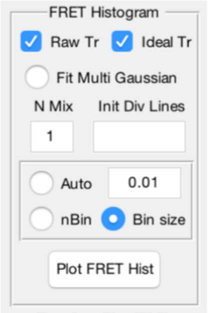

2. In Fig. S1E, TFAM-unbound traces are removed by enabling *'Exclude Steady Low'* = True with *'FRET Threshold'* = 0.2. Replot FRET histograms with this change.
3. In Fig. S2F, the intervals of acceptor bleaching/blinking are removed by enabling *'Exclude Segment'* = Ture with *'FRET Lower Bnd'* = 0.13.

**TDP plot (reproduce Fig. 1E)**

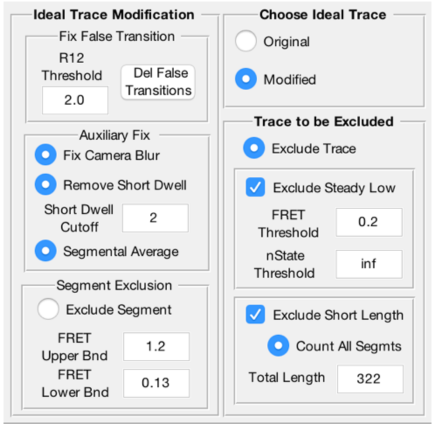


1. Fig. 1E shows TDP after fixing potential noise-induced false transition events. To reproduce it, configure the parameter settings as follows.
   *'Fix False Transition'* panel:
   >> *'R12 Threshold'* = 2.0
   >> Run *'Del False Transitions'*
   *'Auxiliary Fix'* panel:
   >> *'Fix Camera Blur'* = True
   >> *'Remove Short Dwell'* = True
   >> *'Short Dwell Cutoff'* = 2
   >> *'Segment Average'* = True
   *'Segment Exclusion'* panel:
   >> *'Exclude Segment'* = False
   *'Tran Den Plot (TDP)'* panel:
   >> Run *'Plot TDP'*

**Dwell time analysis (reproduce Fig. 1F)**

1. To plot dwell time histograms and perform double exponential function fit for the original traces, configure the parameters as follows.
   *'Ideal Trace Modification'* > *'Auxiliary Fix'* panel:
   >> *'Fix Camera Blur'* = False
   >> *'Remove Short Dwell'* = False
   >> *'Segmental Average'* = False
   *'Choose Ideal Trace'* panel:
   >> *'Original'* = True
   *'Plot'* > *'2-state Model'* panel:
   >> *'High State Threshold'* = 0.43
   >> *'Exclude Start/End Dwell Time'* = True

>> *‘Auto’* = False

>> *‘Drop Short Dwell (Frame) leq’* = True; Enter 2 in the text box

>> *‘Fitting’* = True; Choose exp1 or exp2 in the list box

To plot/analyze the dwell time of lower FRET state (i.e., P-state):

>> *‘Plot Lim (S)’* = 1; *‘Bin Size (Fr)’* = 1; *‘Fit Lim (S)’* = 1

>> Click on ‘Plot Dwell Time’ button

To plot/analyze the dwell time of higher FRET state (i.e., F-state):

>> *‘Plot Lim (S)’* = 5; *‘Bin Size (Fr)’* = 3; *‘Fit Lim (S)’* = 10

**Donor/acceptor channel calibration (reproduce Fig. S5)**

DNA constructs dual-labeled with Cy3 and Atto647, as shown in Fig. S5, are used to calibrate donor and acceptor fluorescence detection channels. To reproduce Fig. S5 and perform the calibration, single-molecule data corresponding to Fig. S5A and S5B should be imported and analyzed.

1. Analysis of DNA with Cy3 located proximal to Atto647 (Fig. S5A):

   ‘*Import smFRET Traces’* panel:

   >> Click on *‘Import Data’*

   >> Go to sample_trace > LSP_Cy3_Atto647 > channel_calibrate folder to choose ‘H-Calibrate_LSP-Cy3-Atto647_ebFRET.mat’ file

   *‘PIFE/FRET Analysis’* panel:

   >> Click on *‘Init smpifefret’* button

   *‘Cal Data 1’* subpanel (left subpanel):

   >> Click on *‘Import Data’* button

   >> Click on *‘Find Events’* button **(Note: This task may take a few minutes)**

   >> Clicking on *‘Plot D/A’* button will display a donor/acceptor 2D heat map, reproducing Fig. S5B

2. Analysis of DNA with Cy3 located distal to Atto647 (Fig. S5B):

   ‘*Import smFRET Traces’* panel:

   >> Click on *‘Import Data’*

   >> Go to sample_trace > LSP_Cy3_Atto647 > channel_calibrate folder to choose ‘L-Calibrate_LSP-Cy3-Atto647_ebFRET.mat’ file

   *‘Cal Data 2’* subpanel (right subpanel):

   >> Click on *‘Import Data’* button

   >> Click on *‘Find Events’* button **(Note: This task may take a few minutes)**

>> Clicking on *'Plot D/A'* button will display donor/acceptor 2D heat map, reproducing Fig. S5E

3. To calibrate channel,

   >> Click on *'Calibrate Channels'* button

   After calibration is done, *'PIFE/FRET Analysis'* panel will look like a screenshot on the right **(Note: The actual values of calibration parameters will be different from the screenshot)**.

   >> Click on *'Plot FRET/PIFE'* button to plot calibrated FRET vs PIFE for the same data used for calibration (i.e., Cal Data 1 and Cal Data 2). This will reproduce Fig. S5C and S5F.

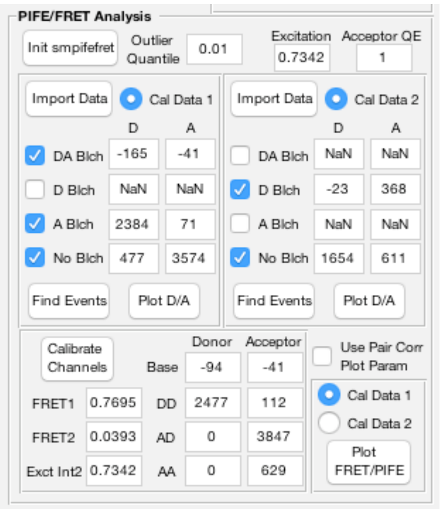


4. The calibration information can be saved and reloaded using *'Save'* and *'Load'* buttons in *'smPifeFret Object'* panel.

**smFRET-smPIFE 2D heat map plot (reproduce Fig. 2F)**

Channel calibration relies on a 2D Gaussian mixture-based clustering algorithm that produces slightly different results every run. The calibration parameters that were used to produce Fig. 2F are therefore provided.

1. Reload the previously saved calibration parameters:

   *'smPifeFret Object'* panel:

   >> Click on *'Load'* button

   >> Go to sample_trace > LSP_Cy3_Atto647 > channel_calibrate folder to choose 'smpifefret_calibration_saved.mat' file

   *'PIFE/FRET Analysis'* panel:

   >> *'Excitation'* = 0.7342

2. Plot FRET vs PIFE 2D heat map for DNA alone:

   *'Import smFRET Traces'* panel:

   >> Click on *'Import Data'*

   >> Go to sample_trace > LSP_Cy3_Atto647 folder to choose 'LSP-Atto647(-41)-Cy3(-12)_ebFRET.mat' file

   *'Segment Exclusion' panel:*

   >> *'Exclude Segment'* = True; *'FRET Lower Bnd'* = 0.12

   (This eliminates acceptor photo-bleaching/-blinking)

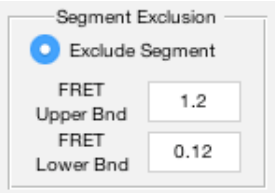

*'Trace To be Excluded'* panel:

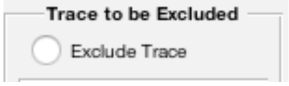


>> *'Exclude Trace'* = False **(Note: 2D heat map plot requires this to be deselected)**

*'Pair Correlation Map*' panel:

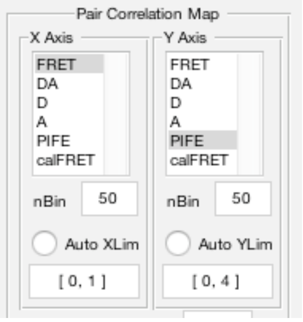


>> '*X Axis'* = FRET; *'Y Axis'* = PIFE

>> *'Auto XLim'* = False; Enter [0, 1] in the text box

>> *'Auto YLim'* = False; Enter [0, 4] in the text box

>> Click on *'Plot Pair Corr'* button

3. Plot FRET vs PIFE 2D heat map for DNA when TFAM is added: Repeat step 2 above with importing 'TFAM_LSP-Atto647(-41)-Cy3(-12)_ebFRET.mat' data file located in sample_trace > LSP_Cy3_Atto647 folder.
