# Supplementary material for: Sequence-specific dynamic DNA bending explains mitochondrial TFAM’s dual role in DNA packaging and transcription initiation
Source: Nat Commun. 2024 Jun 27;15:5446. doi: 10.1038/s41467-024-49728-6 (PMC11211510; doi:10.1038/s41467-024-49728-6)
Supplement: Supplementary file 5 — Reporting Summary [file 41467_2024_49728_MOESM5_ESM.pdf]

nature portfolio

Corresponding author(s): Sang-Hyuk Lee and Smita S Patel

Last updated by author(s): 5/10/2024

Reporting Summary

Nature Portfolio wishes to improve the reproducibility of the work that we publish. This form provides structure for consistency and transparency in reporting. For further information on Nature Portfolio policies, see our Editorial Policies and the Editorial Policy Checklist.

Statistics

For all statistical analyses, confirm that the following items are present in the figure legend, table legend, main text, or Methods section.

n/a	Confirmed	
☐	☒	The exact sample size (*n*) for each experimental group/condition, given as a discrete number and unit of measurement
☐	☒	A statement on whether measurements were taken from distinct samples or whether the same sample was measured repeatedly
☒	☐	The statistical test(s) used AND whether they are one- or two-sided<br>*Only common tests should be described solely by name; describe more complex techniques in the Methods section.*
☒	☐	A description of all covariates tested
☒	☐	A description of any assumptions or corrections, such as tests of normality and adjustment for multiple comparisons
☐	☒	A full description of the statistical parameters including central tendency (e.g. means) or other basic estimates (e.g. regression coefficient) AND variation (e.g. standard deviation) or associated estimates of uncertainty (e.g. confidence intervals)
☒	☐	For null hypothesis testing, the test statistic (e.g. *F*, *t*, *r*) with confidence intervals, effect sizes, degrees of freedom and *P* value noted<br>*Give P values as exact values whenever suitable.*
☒	☐	For Bayesian analysis, information on the choice of priors and Markov chain Monte Carlo settings
☒	☐	For hierarchical and complex designs, identification of the appropriate level for tests and full reporting of outcomes
☒	☐	Estimates of effect sizes (e.g. Cohen's *d*, Pearson's *r*), indicating how they were calculated

Our web collection on statistics for biologists contains articles on many of the points above.

Software and code

Policy information about availability of computer code

Data collection	Single-molecule fluorescence: Nikon NIS Elements; Fluorescence anisotropy: Tecan Spark Cyto; Ensemble FRET: Horiba Fluoromax-4
Data analysis	Hidden Markov Model fitting: ebFRET and vbFRET; Additional single-molecule fluorescence time trace analysis: custom code (https://github.com/shyuklee/smfretpostanalypub)

For manuscripts utilizing custom algorithms or software that are central to the research but not yet described in published literature, software must be made available to editors and reviewers. We strongly encourage code deposition in a community repository (e.g. GitHub). See the Nature Portfolio guidelines for submitting code & software for further information.

Data

Policy information about availability of data

All manuscripts must include a data availability statement. This statement should provide the following information, where applicable:

- Accession codes, unique identifiers, or web links for publicly available datasets
- A description of any restrictions on data availability
- For clinical datasets or third party data, please ensure that the statement adheres to our policy

Data supporting the findings of this study are available in the main text and the Supplementary Materials. Raw microscopy image data and donor-acceptor fluorescence time traces generated in this study have been deposited to Mendeley Data (https://doi.org/10.17632/4whngps32r.1). Source data are provided with this paper.

Research involving human participants, their data, or biological material

Policy information about studies with human participants or human data. See also policy information about sex, gender (identity/presentation), and sexual orientation and race, ethnicity and racism.

Reporting on sex and gender	This is not relevant to our work.
Reporting on race, ethnicity, or other socially relevant groupings	This is not relevant to our work.
Population characteristics	This is not relevant to our work.
Recruitment	This is not relevant to our work.
Ethics oversight	This is not relevant to our work.

Note that full information on the approval of the study protocol must also be provided in the manuscript.

Field-specific reporting

Please select the one below that is the best fit for your research. If you are not sure, read the appropriate sections before making your selection.

☒ Life sciences ☐ Behavioural & social sciences ☐ Ecological, evolutionary & environmental sciences

For a reference copy of the document with all sections, see nature.com/documents/nr-reporting-summary-flat.pdf

Life sciences study design

All studies must disclose on these points even when the disclosure is negative.

Sample size	smFRET studies involve a series of complex computational analysis steps to infer the number of intrinsic molecular states and the transition rates among them from single-molecule time traces. Individual components of smFRET analysis are still active fields of research and there exist a growing number of analysis methods and tools available. However, any statistical method to predetermine a sufficient number of traces and data points for a good estimation of the molecular properties is not well established yet. A recent benchmark study (https://www.nature.com/articles/s41467-022-33023-3) on smFRET analysis demonstrated that simulated 75 traces (59,486 data points) and experimental 19 traces (226,100 data points) corresponding to two-state models led to very similar results among 14 different analyses performed by multiple laboratories. In our smFRET analysis presented in Fig. 1D-F and Fig. 3A-F, we used 420 traces (648,050 data points) for LSP, 88 traces (143,988 data points) for HSP, and 68 traces for NS (89,475 data points). The number of traces and data points for LSP were chosen especially larger than HSP and NS cases because of more than 5-fold slower transition kinetics for LSP. Our smFRET data sizes are comparable to the values used in the previous benchmark study and typical smFRET studies. Moreover, the appearance of two clear populations in the FRET distributions and the very good exponential model fitting results of the dwell time distributions as shown in the figures justify that the sample sizes used in our study are sufficient for reasonable estimation of the relevant parameters regarding the DNA-TFAM conformational dynamics. In addition, we repeated independent experiments with three distinct samples for each DNA sequence to test reproducibility. We incorporated the 95% confidence intervals from the model fitting and the variations in the triplicate data sets into the final uncertainty in the parameter estimates as presented in Supplementary Table 1.
Data exclusions	There exist a population of DNA that is not bound to TFAM. In addition, acceptor photo-bleaching/-blinking produces near zero FRET values. Such types of data are irrelevant to DNA-TFAM interaction; hence, we identified and excluded them for analysis. The details are described in Figure S1.
Replication	All experiments, both single-molecule and ensemble assays, were performed mostly in triplicate or otherwise in duplicate on different days to test data reproducibility. The mean values are overlaid with individual data points in all the bar charts in the manuscript.
Randomization	Typically, smFRET data was acquired from multiple areas of a coverslip and they were all grouped together to produce an analysis result for a certain condition. For the same experimental condition, we repeated multiple independent experiments in different days and analyzed each of them separately to track the variability of the results. Therefore, randomization is not relevant to our data and analysis.
Blinding	smFRET data acquisition could go wrong in many ways and the entire data set would show very heterogeneous behaviors if we included every single data from all attempted experiments to be completely blind. We had to find the patterns that we believed were the primary characteristics of DNA-TFAM interaction from many repeated experiments and analyses.

Reporting for specific materials, systems and methods

We require information from authors about some types of materials, experimental systems and methods used in many studies. Here, indicate whether each material, system or method listed is relevant to your study. If you are not sure if a list item applies to your research, read the appropriate section before selecting a response.

Materials & experimental systems

n/a	Involved in the study
☒	☐ Antibodies
☒	☐ Eukaryotic cell lines
☒	☐ Palaeontology and archaeology
☒	☐ Animals and other organisms
☒	☐ Clinical data
☒	☐ Dual use research of concern
☒	☐ Plants

Methods

n/a	Involved in the study
☒	☐ ChIP-seq
☒	☐ Flow cytometry
☒	☐ MRI-based neuroimaging
